# Supplementary material for: Comparing preferences to evaluations of barrier self-efficacy for two strength training programs in US older adults
Source: PLoS One. 2024 May 9;19(5):e0302892. doi: 10.1371/journal.pone.0302892 (PMC11081341; doi:10.1371/journal.pone.0302892)
Supplement: Attachment — Submitted filename: reviwer comment.docx [file pone.0302892.s002.docx]

Thank you for inviting me to review this paper. This paper is well written and very interesting and important for the readers. However, there are some minor changes required to improve quality of this paper.

**Introduction**

The introduction section should be elaborated by reflecting the significance and novelty of the study.

**Methodology**

What were the exclusion criteria? Who did the survey?

The statistical analysis section should reflect all the tests used.

**Discussion**

In discussion section, focus more on potential implications of your study's findings and add future research direction based on current findings.

**Conclusion**

Clearly state the contributions of your study to the field and highlight any novel aspects of your research. Future work should be mentioned in discussion section.

**Language and Writing Style**

The manuscript requires careful proofreading to eliminate grammatical errors and improve sentence structure. Clarity and precision in language will significantly enhance the manuscript's readability and overall impact.

I appreciate your attention to these matters and look forward to reviewing the revised version of your manuscript.
